# Are There Cognitive and Anxiety Improvements in People With Parkinson’s Disease After Xbox Kinect Training Compared With No Intervention? A Non‐Randomized Controlled Trial

**DOI:** 10.1002/pri.70306

**Published:** 2026-08-10

**Authors:** Ellen Cristine Ferreira da Silva, Wenderson de Souza Morais, Rodrigo Luiz Carregaro, Patricia Azevedo Garcia, Josevan Cerqueira Leal, Felipe Augusto Dos Santos Mendes

**Affiliations:** ^1^ Graduate Program in Rehabilitation Sciences Campus UnB Ceilândia University of Brasília Brasília Brazil

**Keywords:** anxiety, cognition, Parkinson’s disease, virtual reality

## Abstract

**Background and Purpose:**

Cognitive impairment manifests early in Parkinson’s disease (PD), adversely affecting patients’ daily activities and mental health. Utilizing exergames via the Xbox 360 Kinect (XBK) console presents a promising therapeutic avenue to mitigate cognitive decline in PD. This study aimed to assess the efficacy of the XBK system in enhancing cognitive performance and reducing anxiety levels among individuals with PD.

**Method:**

A non‐randomized controlled trial, controlled, single‐blind clinical trial was conducted. Forty‐three PD participants were selected through convenience sampling and non‐randomly assigned to either the XBK experimental group (*n* = 23) or the Control Group (*n* = 23). Participants underwent pre, post, and 30‐day post‐intervention evaluations using the Digit Span Tests, Semantics, Verbal Fluency Test, and Beck Anxiety Inventory. The control group received no intervention, while the experimental group underwent 10 sessions employing four XBK games. This study was approved by the Institutional Ethics Committee of the University of Brasília (Approval No. 2.109.826). Analysis of variance (ANOVA 2 × 3) with Sidak post hoc test and Bonferroni correction was utilized to examine intergroup outcomes.

**Results:**

The XBK‐trained group exhibited enhancements in short‐term memory post‐intervention (*p* = 0.04) and at follow‐up (*p* = 0.01), as well as operational memory post‐intervention (*p* = 0.006) and at follow‐up (*p* = 0.001), although without significant between‐group differences. Although no significant interaction effect was observed (*p* = 0.126), a reduction in anxiety levels was verified up to 30 days post‐training (*p* = 0.03), exclusively within the XBK group, accompanied by a significant between‐group difference (*p* = 0.01), also in follow‐up.

**Discussion:**

Although the intervention did not demonstrate a superior effect compared with the control group, our findings suggest that training with the Xbox 360 Kinect may have the potential to reduce anxiety in individuals with Parkinson's disease. Further research through randomized clinical trials is necessary to validate these findings and evaluate the long‐term benefits of this intervention.

**Trail Registration:**

University of Brasília, CAAE: 12248513.9.0000.0030. N°: 2.109.826 https://ensaiosclinicos.gov.br/rg/RBR‐7h6b8mg.

## 1 Introduction

Cognitive impairment is one of the most common and relevant non‐motor disorders in Parkinson's Disease (PD). This condition compromises the functionality of people with PD, especially before the onset of motor symptoms, in the early stages of PD (Aarsland et al. 2017; De Simone et al. 2023). One relevant cognitive impairment is related to the disordered frontostriatal dysexecutive syndrome, which impacts multitasking and processing speed, toward broader involvement of posterior cortical regions. Such involvement manifests as dysfunction in visuospatial and verbal memory. The prevalence of these impairments affects up to 24% of cases following a PD diagnosis. Their presence may serve as predictive indicators for the development of dementia, worse quality of life, and increased disability, even when motor symptoms are controlled with medication (Aarsland et al. 2017; De Simone et al. 2023; Dirnberger and Jahanshahi 2013; Kletzel et al. 2017; Mills et al. 2020).

Cognitive rehabilitation in individuals with PD includes training to remember and execute intentions, which could be crucial for enhancing social participation in this population (De Simone et al. 2023). Computer games, exclusively designed to meet cognitive demands, provide innovative aspects to cognitive rehabilitation programs (De Simone et al. 2023; Shatil 2013).

In addition to cognitive changes, anxiety disorders are other non‐motor symptoms that affect about 31% of people with PD, often being underdiagnosed and undertreated (Avanzino et al. 2018; Broen et al. 2018; Dissanayaka et al. 2015; Weintraub and Mamikonyan 2019). Anxiety in individuals with PD can exacerbate the severity of motor symptoms, limit functional mobility, elevate the risk of falls, and increase the occurrence of freezing episodes (Avanzino et al. 2018; Broen et al. 2018; Rodriguez‐Oroz et al. 2009).

Exercise therapy was deemed to mitigate anxiety by modulating monoamine levels (e.g., serotonin, dopamine, and norepinephrine) and the stress hormone, cortisol (Alves et al. 2018; Zhang et al. 2023). Moreover, it facilitates neuroprotection via molecular adaptations, sustaining mood and promoting optimal mental health (Alves et al. 2018; Zhang et al. 2023).

Considering the interplay of motor and cognitive impairments in PD, it is recommended that interventions using exercises concurrently address both cognitive and motor demands (Shatil 2013). Virtual reality games training (VRGs) provides simultaneous performance of motor and cognitive tasks while increasing treatment adherence, compared to conventional interventions (Skjæret et al. 2016). In addition, VRGs are promising strategies for training activities that simulate real‐life events, allowing the transfer of cognitive improvement to the patient's daily living (De Simone et al. 2023).

Commercial VRGs, such as the Xbox 360 Kinect (XBK), have the advantage of not requiring controls for interacting with virtual tasks, allowing freedom to move and, consequently, enabling proper attention to the cognitive demands of the games. The XBK sensor is motion‐sensitive and has been considered valid for detecting the movements of people with PD (Adams et al. 2014; Wollersheim et al. 2010) and enriching the therapeutic process (Taylor et al. 2011). Recent evidence suggested that individuals with PD showed improvements in cognitive skills, such as visual‐motor, planning, reaction time, automatic attention, and dual tasking, after training with XBK games (Dantas et al. 2018; Mendes et al. 2015) but also reported safety (Pompeu et al. 2014). Learning after XBK training can also occur for long periods (Pompeu et al. 2012). Notwithstanding, current evidence on the cognitive effects of XBK in people with PD still needs to be consolidated (Mendes et al. 2015; Pompeu et al. 2014, 2012).

There is a lack of evidence regarding the effectiveness of commercial VRG training on mood symptoms in people with PD. To the best of our knowledge, only one study used the VRG of a Nintendo Wii as an intervention for tackling depression in individuals with PD (Zhang et al. 2023). Another study used XBK as a complementary strategy for cognitive function training and anxiety improvement in this population (Marotta et al. 2023). Furthermore, a pilot study investigating the effects of training with Nintendo Wii and XBK in a reduced sample size, on short‐term and working memory, self‐perception of anxiety, and verbal semantic fluency demonstrated no significant improvements in the XBK group (Alves et al. 2018).

Thus, this study aimed to investigate the efficacy of an intervention using XBK games on cognitive functions (short‐term and working memory, executive functions, inhibition control, and semantic verbal fluency) and self‐perception of anxiety in people with PD compared with a control group without any intervention. It is hypothesized that the intervention with XBK will be superior to no intervention by improving cognitive functions and anxiety levels.

## 2 Methods

### 2.1 Study Design

This study is a non‐randomized controlled trial with a blinded outcome assessor. Given the preliminary and exploratory objectives of the study, as well as the logistical and operational constraints related to its implementation, a non‐randomized design was adopted. This preliminary approach was chosen to generate foundational data to inform the design of future randomized clinical trials. Blinding of trainers and participants was not feasible given the characteristics of the intervention and study procedures.

### 2.2 Subjects

The participants were diagnosed by a neurologist and screened by a physiotherapist. Those diagnosed with PD were deemed eligible for this study. All participants were selected through convenience sampling and recruited from a university outpatient clinic specializing in physical therapy for individuals with PD. After performing the baseline assessments, the participants were allocated as soon as they came into the Kinect Group (KG) and Control Group (CG).

Inclusion criteria were: (a) to be in stable treatment with Levodopa; (b) to be classified between 1 and 3 points on the Hoehn and Yahr Scale; (c) to obtain a score of at least 24 points in the Mini‐Mental State Examination; (d) to have at least 4 years of formal education and (e) to have preserved visual and auditory acuities. Exclusion criteria were: (a) to present other neurological disorders or pathological conditions that prevented participation in training; (b) to have previous experience with the XBK exergames; and (c) to attend another rehabilitation program.

All participants signed the informed consent form, and the Institutional Ethics Committee approved the study (University of Brasília, n. 12248513.9.0000.0030).

### 2.3 Procedures

**FIGURE 1:**
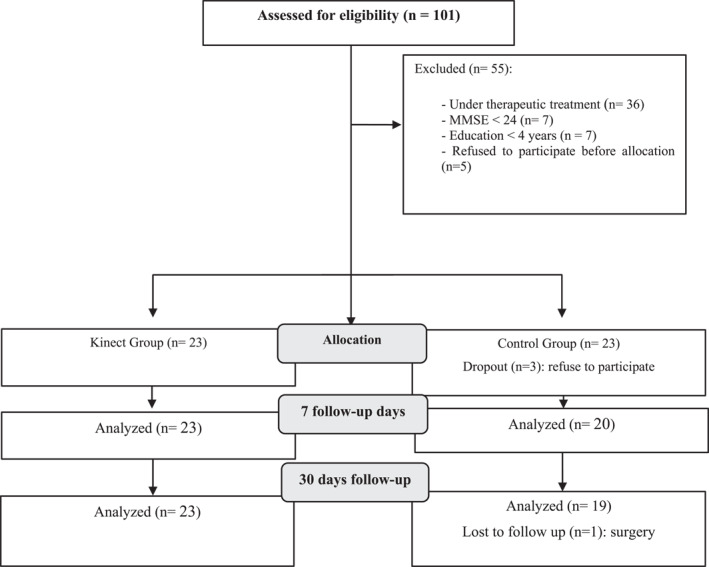
The flowchart of the study.

Participants were allocated to the intervention and control groups in an alternating sequence according to the order of enrollment. As each eligible participant was recruited, they were consecutively assigned to either the intervention or the control group until the desired sample size was achieved. No randomization procedure was used for group allocation. Therefore, although the allocation process followed a predefined sequence, the study design may be subject to selection bias, which should be considered when interpreting the findings. The assessor was trained on the measurement procedures and instruments. All evaluations occurred at the same time of the day. Participants were assessed at three different moments: (1) Baseline, post‐training (until 7 days after the end of the intervention), and follow‐up (30 days after the end of the intervention). The study flowchart is presented in Figure 1.

### 2.4 Primary Outcomes

The primary outcomes were Digit Span Tests and semantic fluency.

We used the Digit Span Tests, which are divided into (a) forward (FDS), which assesses the retention capacity of short‐term memory, and (b) backward (BDS), which assesses the retention capacity of immediate memory and operating memory. Validated for people with PD (Biundo et al. 2013), the tests were adapted, validated, and standardized for Brazilian samples (Figueiredo et al. 1998).

The Semantic Verbal Fluency Test assessed the ability to search and recall semantic memories, executive functions, and language. At the same time, participants articulate as many words as possible about a specified semantic category within a one‐minute timeframe (Silva et al. 2008). It is sensitive to cognitive difficulties commonly seen among people with PD (Koerts et al. 2013). The Scientific Department of Cognitive Neurology and Aging recommended the test for dementia screening (da Silva et al. 2011; Henry and Crawford 2004).

### 2.5 Secondary Outcomes

The secondary outcome was anxiety, measured by the Beck Anxiety Inventory (BAI). This instrument consists of 21 items of a self‐report anxiety scale through which the individual must report the severity of symptoms on a four‐point scale (Baptista and Carneiro 2011). The internal consistency of BAI for use in PD was satisfactory (Cronbach’s = 0.88) as well as its test‐retest reliability (*r* = 0.77; Dissanayaka et al. 2015).

### 2.6 Description of the Interventions

#### 2.6.1 Kinect Group

KG participants received 10 one‐hour individual sessions, twice a week, for five consecutive weeks. During the training sessions, rest intervals were adopted for each participant according to personal needs. For safety, blood pressure, heart rate, and respiratory rate were monitored before, during, and at the end of each training session. Before starting the first session, the trainer provided guidance regarding the objectives of each game and the proper movements to reach them. In each session, games were played three times. Participants received auditory and tactile stimuli and feedback from the trainer in the first two attempts. In the third attempt, participants played without help, except for verbal motivation.

The games were displayed on a screen positioned 2 m away from the participant. The games Hurdles, River Rush, Reflex Ridge, and Light Race from the Kinect Adventures package were used. Details about the game selection method and their respective motor and cognitive demands can be found in the study by Alves et al. (2018).

#### 2.6.2 Control Group

The CG did not receive any intervention. Throughout the study period, participants were instructed to maintain their habitual activities, which had been consistently practiced for a minimum of 6 months prior to the initiation of the study. They should not start or end any physical or cognitive activities during this period.

#### 2.6.3 Sample Size

The sample size calculation was performed in the G*Power version 3.1.9.2 program, considering the a priori repeated measures ANOVA model, within‐between interaction, with an alpha of 5% and statistical power of 80%. The effect size was estimated from a pilot study with participants with the same clinical characteristics as the present study (Alves et al. 2018). The estimated effect size was 0.38, considering the difference between the groups for the Digit Span Tests (Field 2009). The required sample size of 20 participants per group (40 in total) was determined based on the initial calculation. To accommodate an anticipated dropout rate of 15%, the final target sample was increased to 46 participants.

### 2.7 Data Analysis

The Shapiro‐Wilk test confirmed the data normality. The Repeated Measures Analysis of Variance (ANOVA 2 × 3) was applied with the Sidak post hoc test and Bonferroni correction, considering the groups (KG and CG) and evaluation times (baseline, post‐training, and follow‐up) as factors for parametric variables. Mauchly’s sphericity test was considered and, if necessary, the corrected value of Greenhouse‐Geisser was adopted.

The Effect Size was calculated by Cohen’s *d*, where *d* = M1 − M2/*s* (mean difference between, M1 − M2, divided by the pooled standard deviation—s); being: Cohen *d* ≥ 0.2 small; *d* ≥ 0.5 medium; *d* ≥ 0.8 large (Field 2009). The significance was 5% (*p* < 0.05), with a 95% confidence interval.

## 3 Results/Findings

Figure 1 depicts the flowchart of the study, which shows the recruitment process and dropouts.

A total of 101 participants were assessed for eligibility. After application of the exclusion criteria, 46 participants were included in the study and allocated to the KG (23 participants—8 women) or the CG (23 participants—8 women). However, three participants in the CG withdrew from the study after the first intervention. Their data were excluded from the analysis. No adverse effects were reported. Baseline characteristics are presented in Table 1, and no significant differences between groups were observed at baseline.

**TABLE 1:** Baseline characteristics of the participants.

Characteristics	Kinect group (*n* = 23)	Control group (*n* = 23)	*p* value
Age (years) mean (SD)	63.8 (12.1)	62.9 (10.7)	0.80
Gender (men/women) N/N	15/8	15/8	0.58^a^
Education (years) mean (SD)	12.23 (4.2)	11.24 (4.4)	0.48
H&Y stages mean (SD)	1.95 (0.7)	1.94 (0.8)	0.95
MMSE (score) mean (SD)	28.1 (1.3)	28.2 (1.9)	0.92

*Note:* Student’s t test.

Abbreviations: H&Y, Hoehn e Yahr scale; MMSE, Mini‐Mental State Exam.

^a^Chi‐squared test.

Repeated‐measures ANOVA was used to examine the effects of time and group on cognitive outcomes (Table 2). For verbal fluency, no effect of time (*F*(1.613, 58.060) = 1.158, *p* = 0.312), group × time interaction (*F*(1.613, 58.060) = 0.804, *p* = 0.428), or main effect of group (*F*(1, 36) = 1.061, *p* = 0.310) was observed. For BDS, a significant effect of time (*F*(2, 72) = 7.881, *p* = 0.001) and group × time interaction (*F*(2, 72) = 4.191, *p* = 0.019) were found, with improvements observed only in the Kinect group. Similarly, FDS showed a significant effect of time (*F*(1.479, 53.235) = 4.129, *p* = 0.032) and group × time interaction (*F*(1.479, 53.235) = 4.858, *p* = 0.019), again with improvements restricted to the Kinect group.

**TABLE 2:** Pre‐post and pre‐follow‐up training changes in primary outcomes in the Kinect group (KG) and control group (CG).

Outcomes	Group	Pre	POST	p*	ES	FU	p**	ES
Verbal fluency	KG	16.5 (5.4)	16.2 (5.7)	0.99	0.23	16.2 (4.2)	0.98	0.06
	CG	15.5 (6.6)	13.5 (3.9)	0.43	0.37	15.3 (6.1)	0.99	0.03
	p*	0.61	0.11			0.57		
FDS	KG	9.9 (2.3)	11.5 (2.6)	0.04	0.65	11.0 (2.5)	0.01	0.46
	CG	12.3 (1.8)	12.3 (2.0)	0.99	0	11.8 (1.8)	0.64	0.28
	p*	0.06	0.32			0.34		
BDS	KG	5.9 (2.8)	7.3 (2.7)	0.006	0.50	7.7 (2.6)	0.001	0.66
	CG	6.9 (1.9)	7.6 (2.0)	0.46	0.35	7.0 (2.7)	0.99	0.04
	p*	0.22	0.75			0.42		

*Note:* Sidak's pos hoc test: * post × pré; ** FU × pré.

Abbreviations: BDS, Backward Digit Span Test; ES, Effect Size; FDS, Forward Digit Span Test.

For anxiety symptoms measured by the Beck Anxiety Inventory (Table 3), no significant effect of time (*F*(1.637, 58.939) = 2.435, *p* = 0.106) or group × time interaction (*F*(1.637, 58.939) = 2.226, *p* = 0.126) was observed. However, between‐group differences were found at post‐training (*F*(1, 36) = 5.309, *p* = 0.027) and follow‐up (*F*(1, 36) = 6.856, *p* = 0.013). These differences indicate that the groups did not present equivalent anxiety scores at those points at that time, although this pattern cannot be interpreted as an effect of the intervention in the absence of a significant interaction.

**TABLE 3:** Pre‐post and pre‐follow‐up training changes in secondary outcome in the Kinect group (KG) and control group (CG).

Outcome	Group	Pre	Post	p*	ES	Follow up	p*	ES
BAI	KG	10.9 (7.7)	8.4 (5.0)	0.12	0.40	8.4 (5.1)	0.03	0,40
	CG	13.1 (9.0)	12.3 (5.2)	0.89	0.11	13.8 (7.7)	0.89	0.08
	p*	0.41	0.02			0.01		

*Note:* Sidak's pos hoc Test: * post × pré; ** FU × pré.

Abbreviations: BAI, Beck Anxiety Inventory; ES, Effect Size.

## 4 Discussion

The current investigation examined the effects of an intervention with XBK games on cognitive performance and anxiety levels in people with PD compared with a non‐intervention group.

Significant intragroup differences were observed in the XBK intervention group, particularly in short‐term memory, and operational memory, when evaluated by FDS and BDS, respectively, beyond the anxiety levels. Although no significant intergroup differences have been identified, moderate effect sizes among post‐training evaluations versus baseline were found in the KG compared with CG in those outcomes. The Verbal Fluency Test did not improve after training, probably because it is not sensitive to the effects of exergaming training, as shown in a previous study (Alves et al. 2018).

Following the VR training protocol outlined in this study, improvements in both types of memory were observed and sustained even after 30 days without intervention. Although no significant difference was found between the study groups, using exergames contributed to enhancing memory abilities, as evidenced by the improvement between pre‐ and post‐evaluation moments, exclusively in the XBK group. Although speculative, this improvement could be attributed to the cognitive demands of the used exergames, which demand players to memorize essential information for completing virtual tasks (Yang et al. 2023). This cognitive engagement facilitates goal achievement and leads to improved performance and the repetition of the memorization tasks in each training session possibly also favored these results (Marotta et al. 2023). These observations could be related to the VRG used in the present study, which required participants to memorize, for example, routes that should be followed, obstacles that should be avoided, and sequences of movements that needed to be performed so that the objectives of each game could be achieved. Tasks that required sustained attention to stimuli, visual and auditory cues that were presented during training, beyond the constant offer of extrinsic feedback of results and performance, during the games, possibly motivated the participants to stay focused throughout the training period, further favoring their ability to memorize (Butler and Willett 2010).

Regarding the assessment of anxiety, the KG participants showed an intergroup difference in anxiety symptoms that lasted up to 30 days after the end of training. This suggests that while the intervention group improved, the control group did not worsen, but the change over time was not different between the groups. This reduction was notable despite the minimal clinically important difference (MCID) for the Beck Anxiety Inventory being 8.8 points (El Morr et al. 2024). Although the design does not allow firm conclusions about mechanisms, the literature suggests that exercises are effective for treating anxiety when performed 2 to 5 times a week, for 5–12 weeks, a dose similar to that used in this study (Wipfli et al. 2008). From a conceptual standpoint, this pattern may align with the offer of auditory and visual stimuli and feedback by the games used. Moreover, the challenges imposed by their tasks may have contributed to the participants being motivated to gradually perform wider body movements previously avoided due to fear of falling (Marotta et al. 2023). A potential contributing factor could be the opportunities to train daily cognitive and physical demands in a virtual environment, such as decision‐making and dual‐task performance. Such training can be acquired and may positively influence daily activities (De Simone et al. 2023; Lu et al. 2022; Mendes et al. 2012). The repeated practice of these movements during training facilitated an improved recognition of their physical capabilities, increased confidence in performing movements, and reduced anxiety levels (Garcia‐Agundez et al. 2019).

### 4.1 Limitations

The volunteers in the present study had HY between 1 and 2. Thus, caution must be exercised in generalizing our results for more severe clinical conditions in PD. Furthermore, the study was not randomized, requiring future randomized clinical trials to better validate the results. Although significant intragroup differences were observed in the assessment of Short‐Term Memory and Operational Memory, it is essential to highlight that no statistically significant differences were observed between the study groups, which does not confirm the effectiveness of this type of training to improve these capabilities in this population. In this sense, the non‐significant interaction for anxiety limits the strength of their conclusion regarding its efficacy.

## 5 Implications of Physiotherapy Practice

Our study demonstrated that a 10‐session intervention utilizing four commercial exergames from the Kinect Adventures game package on the Xbox 360 Kinect was associated with improvements in Short‐Term Memory and Operational Memory within the intervention group. Besides, although participants in the intervention group presented lower anxiety scores at some time points, no significant group × time interaction was detected. Therefore, these differences cannot be interpreted as evidence of an intervention‐driven reduction in anxiety.

Given these limitations, the potential clinical relevance of exergame‐based physiotherapy should be considered cautiously. While the intervention appears feasible and may support certain cognitive domains, its effects on anxiety remain inconclusive. Further investigation is needed to optimize exergame protocols and assess their long‐term efficacy and practical implementation in clinical settings.

## Author Contributions

Ellen Cristine Ferreira da Silva contributed to the interpretation of results, contributed to manuscript writing, and critically revised the manuscript for important intellectual content. Wenderson de Souza Morais conceptualized and designed the study, and data acquisition and wrote the initial draft of the manuscript. Rodrigo Luiz Carregaro contributed to the interpretation of results, provided statistical analysis, and critically revised the manuscript for important intellectual content. Patricia Azevedo Garcia contributed to the interpretation of results and critically revised the manuscript for important intellectual content. Josevan Cerqueira Leal contributed to the interpretation of results and critically revised the manuscript for important intellectual content. Felipe Augusto Dos Santos Mendes conception and design of the work. Analyzed the data, provided statistical analysis, and approved the final version to be published.

## Funding Statement

This research was supported by the “Fundação de Apoio à Pesquisa do Distrito Federal (FAPDF)” “(Grant 193.000655/2015) and the “Coordenação de Aperfeiçoamento de Pessoa de Nível Superior”(Grant 88887.820655/2023‐00)”. The funders played no role in the study design, data collection, analysis, or manuscript preparation.

## Ethics Statement

All participants signed the informed consent form, and the Institutional Ethics Committee approved the study (University of Brasília, CAAE: 12248513.9.0000.0030. N°: 2.109.826). Available in Supporting Information S1.

## Consent

See Supporting Information S2.

## Conflicts of Interest

The authors declare no conflicts of interest.

## Permission to Reproduce Material From Other Sources

We declare that, upon acceptance of the article, *Physiotherapy Research International* will hold the rights to all content, including any reproduced copyrighted material for which permissions have been obtained. Copyright will become the exclusive property of the journal.

## Supporting information


Supporting Information S1

Supporting Information S2

## Data Availability

The data that support the findings of this study are available from the corresponding author upon reasonable request.
