# Supplementary material for: Are There Cognitive and Anxiety Improvements in People With Parkinson’s Disease After Xbox Kinect Training Compared With No Intervention? A Non‐Randomized Controlled Trial
Source: Physiother Res Int. 2026 Aug 10;31(4):e70306. doi: 10.1002/pri.70306 (PMC13457693; doi:10.1002/pri.70306)
Supplement: Supplementary file 1 [file PRI-31-e70306-s002.pdf]

PARECER CONSUBSTANCIADO DO CEP

DADOS DO PROJETO DE PESQUISA

Título da Pesquisa: Aprendizado motor e modificações em aspectos clínicos e funcionais após treinamento baseado em realidade virtual em pacientes com a Doença de Parkinson e idosos saudáveis: efeitos das demandas dos jogos de vídeo games.

Pesquisador: FELIPE AUGUSTO DOS SANTOS MENDES

Área Temática:

Versão: 2

CAAE: 12248513.9.0000.0030

Instituição Proponente: PROGRAMA DE PÓS-GRADUAÇÃO EM CIÊNCIAS E TECNOLOGIAS EM

Patrocinador Principal: Financiamento Próprio

DADOS DO PARECER

Número do Parecer: 201.670

Data da Relatoria: 25/02/2013

Apresentação do Projeto:

Os objetivos deste estudo serão investigar e comparar os efeitos de um treinamento cognitivo-motor baseado em realidade virtual por meio de jogos dos vídeo games Nintendo Wii® e/ou Xbox Kinect®, com diferentes demandas motoras e cognitivas, sobre a aprendizagem, equilíbrio, marcha, função de membros superiores, aspectos cognitivos, aspectos funcionais e percepção de qualidade de vida de pacientes com Doença de Parkinson (DP) e idosos saudáveis, da mesma faixa etária. Esse treinamento será também comparado a outro realizado em ambiente real por meio de técnicas comumente utilizadas em sessões de fisioterapia convencional para ambas as populações. Para isso, será conduzido um estudo clínico, randomizado, cego, longitudinal e controlado. Vinte pacientes em estágio de inicial a moderado da DP, segundo a escala de Hoehn e Yahr, e vinte idosos saudáveis participarão do estudo e serão inicialmente avaliados em testes de equilíbrio, marcha, função de membros superiores, funcionalidade em atividades de vida diária, cognição e percepção da qualidade de vida. Após as avaliações iniciais, os participantes serão randomicamente distribuídos em 4 grupos constituídos por 12 participantes cada: Grupo Experimental DP, Grupo Controle DP, Grupo Experimental Idoso e Grupo Controle Idoso. Os Grupos Experimentais serão submetidos a um treinamento que compreenderá de 10 a 14 sessões de prática em 10 diferentes jogos dos vídeo games Nintendo Wii® e/ou Xbox Kinect®, realizadas

de 2 a 3 vezes por semana, enquanto os Grupos Controles serão submetidos a um treinamento com o mesmo número de sessões, que serão constituídas por exercícios comuns em sessões de fisioterapia convencional e baseados nos movimentos dos jogos. Vinte e quatro horas e 30 dias após o término dos treinamentos, todos participantes serão novamente avaliados pelos mesmos testes realizados antes do treino para verificação dos seus efeitos imediatos e de retenção, respectivamente. O aprendizado dos participantes dos Grupos Experimentais em cada um dos jogos praticados será baseado em seus desempenhos representados pelas pontuações obtidas em cada uma das sessões de treinamento, na avaliação pós-treinamento imediata e na retenção. Os resultados obtidos serão analisados por meio de testes estatísticos adequados para que se possam verificar os efeitos dos treinamentos propostos sobre a aprendizagem, no caso dos grupos experimentais, e sobre a melhora do equilíbrio, da marcha, da função de membros superiores, da funcionalidade nas atividades de vida diária, da cognição e da percepção da qualidade de vida dos pacientes com DP, comparando-os com os idosos saudáveis. Acredita-se que a capacidade de pacientes com DP em melhorar e reter o desempenho após o treinamento nos vídeo games dependerá grandemente das demandas dos jogos envolvidos, reiterando a importância da seleção adequada dos jogos com proposta de reabilitação e que a prática nesse tipo de treino resultará em melhora das alterações clínicas e funcionais apresentadas por essas populações, que será igual ou superior àquelas obtidas pelo treinamento convencional.

Objetivo da Pesquisa:

objetivos primários deste estudo serão:

1. Investigar a aprendizagem de pacientes com DP em jogos de vídeo games Nintendo Wii® e/ou Xbox Kinect® com diferentes demandas motoras e cognitivas, por meio das modificações do desempenho, comparando-a com a aprendizagem de indivíduos saudáveis da mesma faixa etária.

2. Comparar os efeitos de dois programas de treinamento, um deles realizado em ambiente virtual por meio de jogos dos vídeo games e o outro realizado em ambiente real, sobre o equilíbrio, marcha, função de membros superiores, funcionalidade nas atividades de vida diária, cognição e qualidade de vida dos participantes.

3.2 OBJETIVO SECUNDÁRIO:

1. Comparar as modificações de desempenho inter sessões de treino em pacientes com DP e idosos saudáveis, em diferentes jogos dos vídeo games Nintendo Wii® e/ou Xbox Kinect®.

2. Comparar as modificações de desempenho mantidas após 24 horas e 30 dias sem treinamento

em
pacientes com DP e idosos saudáveis, em diferentes jogos dos vídeo games Nintendo Wii® e/ou Xbox Kinect®
3. Investigar a influência das diferentes demandas motoras e cognitivas dos jogos dos vídeo games Nintendo Wii® e/ou Xbox Kinect® nas modificações de desempenho pelo treinamento, em pacientes com DP e idosos saudáveis.
4. Comparar os efeitos de dois tipos de treinamento, um realizado em ambiente real e outro em ambiente virtual, sobre as seguintes capacidades funcionais de pacientes com DP: Equilíbrio, marcha, função de membros superiores, funcionalidade nas atividade de vida diária, cognição e percepção da qualidade de vida.

Avaliação dos Riscos e Benefícios:

Não há riscos esperados para os participantes deste estudo. Contudo, apesar das sessões de treinamento virtual e avaliações das pontuações obtidas nos jogos serem realizadas de forma individual, não sendo permitida a observação dos treinamentos por outros participantes, a utilização de um recurso terapêutico envolvendo jogos virtuais que determinam o grau de sucesso da tentativa via pontuação obtida, poderá trazer algum sentimento de frustração e desânimo aos participantes que não obtiverem êxito. Considera-se uma possibilidade pequena para ocorrência desse risco, entretanto, uma vez que os participantes terão preenchido o critério de inclusão de pontuação máxima de cinco na Escala de Depressão em Geriatria de 15 itens (Almeida e Almeida, 1999), o que exclui a possibilidade dos mesmos serem deprimidos.
São esperados benefícios moderados, tais como melhora do equilíbrio, da marcha, da função de membros superiores, da funcionalidade, da cognição e da percepção da qualidade de vida dos participantes submetidos ao treinamento com uso dos vídeogames.

Comentários e Considerações sobre a Pesquisa:

Os pesquisadores propõem um estudo clínico, randomizado, cego, longitudinal e controlado. Vinte pacientes em estágio de inicial a moderado da DP, segundo a escala de Hoehn e Yahr, e vinte idosos saudáveis participarão do estudo e serão inicialmente avaliados em testes de equilíbrio, marcha, função de membros. Na segunda submissão do projeto, os pesquisadores consideram, mesmo que de caráter remoto, a possibilidade de possível sensação de frustação por parte dos sujeitos da pesquisa, esclarecendo que essa ocorrência se manifesta será de perfil restrito e que não haverá uma percepção comparativa entre os participantes, o que poderia incrementar essa percepção. Essa consideração foi incluída também no TCLE oferecendo maior clareza aos participantes da pesquisa quanto a possibilidade.Adicionalmente, o projeto compõe uma atividade de extensão do curso de fisioterapia da Faculdade de Ceilândia da UnB, onde a prática dessas

atividades lúdicas são rotineiras.Os currículos Lattes dos pesquisadores acompanham o presente pedido, oferecendo condições satisfatórias para condução do projeto. O projeto encontra-se descrito de maneira satisfatória agora atendendo às solicitações de complementação sugeridas por este CEP, especialmente na possibilidade de oferta de riscos aos sujeitos. O cronograma apresentado é compatível com a pretensão de análise. Houve correção nesta submissão dos custos do projeto que estarão relacionados aos equipamentos que serão utilizados na pesquisa e no material de suporte.O pesquisador encaminhou o termo de Consentimento livre e esclarecido, acrescido das sugestões deste CEP, que se encontra estruturado de acordo com a resolução 196/96 CNS/MS. O termo de concordância institucional para o desenvolvimento da pesquisa encontra-se satisfatório. Com isso, entende-se que a documentação apresentada: proposta de pesquisa; projeto; cronograma de atividades, atendem aos requisitos estabelecidos na resolução 196 de 09/10/1996 do Conselho Nacional de Saúde do Ministério da Saúde.

Considerações sobre os Termos de apresentação obrigatória:

O pesquisador encaminhou o termo de Consentimento livre e esclarecido que se encontra estruturado de acordo com a resolução 196/96 CNS/MS, encontrando-se na segunda análise com a consideração a respeito de possíveis reações psicológicas adversas por parte dos sujeitos da pesquisa (frustração e desânimo) durante as atividades com jogos frente a possibilidade de não êxito nos escores obtidos, oferecendo a necessária clareza quanto aos desdobramentos, mesmo que remotos da participação de cada indivíduo. O termo de concordância institucional para o desenvolvimento da pesquisa encontra-se satisfatório

Recomendações:

Considerando a possibilidade, mesmo que remota, de manifestações adversas frente a participação na presente pesquisa sugere-se atenção e rigor quanto à suspensão imediata das atividades aos indivíduos que manifestarem comportamento inesperado.

Conclusões ou Pendências e Lista de Inadequações:

Considerando o atendimento das solicitações deste CEP, bem como, maior esclarecimento tanto no delineamento da pesquisa relativo aos possíveis desdobramentos das práticas vivencia virtual quanto no Termo de consentimento, indica-se parecer favorável à realização do projeto.

Situação do Parecer:

Aprovado

Necessita Apreciação da CONEP:

Não

Considerações Finais a critério do CEP:

O documento de aprovação estará disponível na secretaria do cep a partir do dia 28/02/13.

BRASILIA, 21 de Fevereiro de 2013

Assinador por:
Natan Monsores de Sá
(Coordenador)

Endereço: Facul. de Ciências da Saúde-Campus Darcy Ribeiro
Bairro: Lago Sul **CEP:** 70.910-900
UF: DF **Município:** BRASILIA
Telefone: (61)3307-2113 **Fax:** (61)3307-3799 **E-mail:** cepfs@unb.br
