# Supplementary material for: Are There Cognitive and Anxiety Improvements in People With Parkinson’s Disease After Xbox Kinect Training Compared With No Intervention? A Non‐Randomized Controlled Trial
Source: Physiother Res Int. 2026 Aug 10;31(4):e70306. doi: 10.1002/pri.70306 (PMC13457693; doi:10.1002/pri.70306)
Supplement: Supplementary file 2 [file PRI-31-e70306-s001.docx]

**APPENDIX 2.** **Patient consent statement**

**Termo de Consentimento Livre e Esclarecido – TCLE (Grupo Experimental)**

O (a) Senhor (a) está sendo convidado (a) a participar do projeto: Efeitos motores e cognitivos de pacientes com a doença de Parkinson após treinamento baseado em realidade virtual. O objetivo desta pesquisa é: verificar e comparar os efeitos de dois programas de treinamento virtual sobre o desempenho motor e cognitivo de pacientes com doença de Parkinson.

O (a) senhor (a) receberá todos os esclarecimentos necessários antes e no decorrer da pesquisa e lhe asseguramos que seu nome não aparecerá, sendo mantido o mais rigoroso sigilo através da omissão total de quaisquer informações que permitam identificá-lo (a).

Você fará parte de um programa de fisioterapia cujas atividades serão baseadas em tarefas virtuais que exigem movimentos de todo o corpo para serem realizadas. Este programa compreenderá dez sessões de prática. Além desse treinamento, serão realizadas avaliações da sua forma de andar e da capacidade de seu cérebro a depender de suas necessidades. As avaliações serão realizadas em três períodos: antes do início do treinamento e 7 e 30 dias após o treinamento. Todos os procedimentos serão realizados na Faculdade Ceilândia da UnB, em data previamente combinada. Será gasto um tempo de aproximadamente uma hora e trinta minutos para os dias de avaliação e de uma hora para os dias de treinamento. Informamos que o (a) Senhor (a) pode se recusar a responder (ou participar de qualquer procedimento) qualquer questão que lhe traga constrangimento, podendo desistir de participar da pesquisa em qualquer momento sem nenhum prejuízo para o (a) senhor (a). Sua participação é voluntária, isto é, não há pagamento por sua colaboração.

Os resultados da pesquisa serão divulgados na Instituição Faculdade de Educação Física da UnB, podendo ser publicados posteriormente. Os dados e materiais utilizados na pesquisa ficarão sobre a guarda do pesquisador.

Se o (a) Senhor (a) tiver qualquer dúvida em relação à pesquisa, por favor telefone para: Wenderson de Souza Morais: (--) ------ -------.

Este documento foi elaborado em duas vias, uma ficará com o pesquisador responsável e a outra com o sujeito da pesquisa.

| Nome / assinatura |  | Pesquisador Responsável |
| --- | --- | --- |

Brasília, ___ de __________ de ____

**Termo de Consentimento Livre e Esclarecido – TCLE (Grupo controle)**

O (a) Senhor (a) está sendo convidado (a) a participar do projeto: Efeitos motores e cognitivos de pacientes com a doença de Parkinson após treinamento baseado em realidade virtual. O objetivo desta pesquisa é: verificar e comparar os efeitos de dois programas de treinamento virtual sobre o desempenho motor e cognitivo de pacientes com doença de Parkinson.

O (a) senhor (a) receberá todos os esclarecimentos necessários antes e no decorrer da pesquisa e lhe asseguramos que seu nome não aparecerá, sendo mantido o mais rigoroso sigilo através da omissão total de quaisquer informações que permitam identificá-lo (a).

Você fará parte de um grupo que será encaminhado para um programa de reabilitação de

pacientes com Parkinson após o período de realização do estudo. Serão realizadas avaliações da sua forma de andar e da capacidade de seu cérebro a depender de suas necessidades. As avaliações serão realizadas em três períodos: antes do início do período de treinamento e 7 e 30 dias após esse período. Todos os procedimentos serão realizados na Faculdade Ceilândia da UnB, em data previamente combinada. Será gasto um tempo de aproximadamente uma hora e trinta minutos para os dias de avaliação. Informamos que o (a) Senhor (a) pode se recusar a responder (ou participar de qualquer procedimento) qualquer questão que lhe traga constrangimento, podendo desistir de participar da pesquisa a qualquer momento sem nenhum prejuízo para o (a) senhor (a). Sua participação é voluntária, isto é, não há pagamento por sua colaboração.

Os resultados da pesquisa serão divulgados na Instituição Faculdade de Educação Física

da UnB, podendo ser publicados posteriormente. Os dados e materiais utilizados na pesquisa ficarão sobre a guarda do pesquisador.

Se o (a) Senhor (a) tiver qualquer dúvida em relação à pesquisa, por favor telefone para:

Wenderson de Souza Morais: (--) ------- -------.

Este documento foi elaborado em duas vias, uma ficará com o pesquisador responsável e

a outra com o sujeito da pesquisa.

| Nome / assinatura |  | Pesquisador Responsável |
| --- | --- | --- |

Brasília, ___ de __________ de ____
